# The Transcription Factor Zfx Regulates Peripheral T Cell Self-Renewal and Proliferation

**DOI:** 10.3389/fimmu.2018.01482

**Published:** 2018-07-04

**Authors:** Matthew R. Smith-Raska, Teresita L. Arenzana, Louise M. D’Cruz, Alireza Khodadadi-Jamayran, Aristotelis Tsirigos, Ananda W. Goldrath, Boris Reizis

**Affiliations:** ^1^Department of Microbiology and Immunology, Columbia University Medical Center, New York, NY, United States; ^2^Division of Biological Sciences, University of California San Diego, La Jolla, CA, United States; ^3^Applied Bioinformatics Laboratories, NYU School of Medicine, New York, NY, United States; ^4^Department of Pathology, NYU School of Medicine, New York, NY, United States

**Keywords:** T cell self-renewal, T cell homeostasis, homeostatic proliferation, hematopoietic stem cell, T cell stress response

## Abstract

Peripheral T lymphocytes share many functional properties with hematopoietic stem cells (HSCs), including long-term maintenance, quiescence, and latent proliferative potential. In addition, peripheral T cells retain the capacity for further differentiation into a variety of subsets, much like HSCs. While the similarities between T cells and HSC have long been hypothesized, the potential common genetic regulation of HSCs and T cells has not been widely explored. We have studied the T cell-intrinsic role of *Zfx*, a transcription factor specifically required for HSC maintenance. We report that T cell-specific deletion of *Zfx* caused age-dependent depletion of naïve peripheral T cells. *Zfx*-deficient T cells also failed to undergo homeostatic proliferation in a lymphopenic environment, and showed impaired antigen-specific expansion and memory response. In addition, the invariant natural killer T cell compartment was severely reduced. RNA-Seq analysis revealed that the most dysregulated genes in Zfx-deficient T cells were similar to those observed in Zfx-deficient HSC and B cells. These studies identify *Zfx* as an important regulator of peripheral T cell maintenance and expansion and highlight the common molecular basis of HSC and lymphocyte homeostasis.

## Introduction

A hallmark of acquired immunity is the ability to mount a highly specific response to a wide variety of foreign antigens. Central to this process is a tightly regulated, complex program of T cell homeostasis that involves coordination of cell survival, expansion, differentiation, and death in a number of different contexts. A variety of regulatory mechanisms ensure that the appropriate rare T cell responds to an antigen and maintains a memory of antigen exposure, while the remaining T cell pool actively represses both inappropriate hyperactivation as well as death while maintaining a diverse T cell compartment for the entire life of the organism.

Upon completion of development in the thymus, a T cell enters the periphery as a quiescent, “naïve” cell that expresses CD62L (1). Long-term survival of naïve T cells is mediated by continuous stimulation by self peptide–MHC complexes as well as the cytokine IL-7 (2). Over the life of an organism, naïve T cells are continuously lost due to cell death, differentiation, and migration. Mathematical modeling and experimental data have revealed that, as an organism ages and thymic T cell production decreases, peripheral T cell numbers are maintained in the periphery through a chronic mechanism of replenishment by proliferation (3, 4). In cases of excessive loss of T cells, which can occur following chemotherapy or HIV infection, the remaining T cells respond by proliferating to regenerate a full T cell compartment in a process known as “homeostatic proliferation” (5, 6). Similar to the everyday process of peripheral T cell survival, homeostatic proliferation is driven by self peptide–MHC complexes and IL-7; after this process is complete the T cells acquire the phenotypic and functional properties of a memory cell, known as a “memory phenotype cell” (7, 8, 9).

Recognition of a foreign antigen stimulates a T cell to activate a complex program of expansion and differentiation into an effector cell. Upon stimulation by an antigen, a T cell divides ~14 times, generating more than 10 million antigen-specific cells and increasing its biomass by thousands (9, 10). After the antigen has been cleared, most effector cells die by apoptosis; a small minority persists indefinitely as memory cells, which are maintained in the periphery by IL-7 and IL-15. Similarly to conventional T cells, invariant natural killer T cell (iNKT) cells develop from T cell progenitors in the thymus during which they must successfully mediate multiple stages of expansion and differentiation (11, 12, 13).

T cells are unique among mature, differentiated cell types because they have stem cell-like properties of self-renewal and multipotency. Indeed, T cells have the capacity to maintain constant numbers independent of progenitor-based production through a process of self-renewal. This is evident as T cell numbers remain constant after the thymus involutes early in mammalian life, and after T cells are transferred into a lymphopenic mouse and the final number of cells generated is independent of the number transferred (14, 15). In an elegant display of their multipotency, a single CD8^+^ T cell was shown to be capable of generating the entire diversity of effector and memory CD8^+^ T cells (16). Furthermore, much like stem cells, memory T cells undergo self-renewal in the bone marrow “niche,” and have a gene expression pattern more similar to hematopoietic stem cells (HSC) than naïve or effector T cells, while expressing telomerase much like stem cells (17–21). Indeed, CD4^+^ and CD8^+^ antigen-specific T cells can be detected up to 75 years after vaccination in humans (22). Recent studies have revealed a population of stem cell-like memory T cells that are capable of self-renewal as well as proliferation and differentiation into effector cells in response to antigen re-exposure (23, 24). There have also been descriptions of T cells with stem cell-like properties in graft versus host disease, as well as the existence of quiescent memory CD8^+^ T cells that express the stem cell gene c-kit and are resistant to chemotherapy, much like HSC (25, 26). Recently, the histone methyltransferase *Suv39h1* was shown to be essential for silencing stem cell-related genes in CD8^+^ effector T cells (27).

*Zfx* is a zinc finger transcription factor located on the X chromosome that is strongly conserved throughout vertebrate evolution. *Zfx* is expressed consistently across all tissues and cells in an organism, as well as during the various stages of cell development. *Zfx* is essential for survival of mature recirculating B cells, HSCs, and embryonic stem cells (ESC) (28–30). In addition, multiple recent reports have revealed that *Zfx* is overexpressed in multiple different human cancers, including glioblastoma, hepatic cell carcinoma, and renal cell carcinoma and is required in mice for the initiation and maintenance of leukemia (31–33).

Despite the functional similarities between HSCs and mature T cells, support for genetic similarities has thus far been sparse. The self-renewal defects in *Zfx*-null HSC, coupled with the concept that T cells display self-renewal and multi-potency, led us to study the role of *Zfx* in mature T lymphocytes. Here, we show that *Zfx*-deficient peripheral T cells fail to persist over time. In addition, loss of *Zfx* causes a defect in homeostatic proliferation and expansion upon antigen stimulation, as well as memory T cell expansion after antigen re-exposure. Furthermore, *Zfx* deficiency inhibits the development of iNKT cells. Gene expression analysis reveal common transcriptional abnormalities shared with *Zfx*-deficient HSC, suggesting a common mechanism of function.

## Materials and Methods

### Mice

Conditional knockout (CKO) (*Zfx*^flox/y^ Cre^+^) mice were generated by crossing pure 129/SvEv *Zfx*^flox/flox^ females with CD4-Cre^+^ (mixed 129/B6) (34) or Rosa26-CreER^+^ (35) transgenic males. To generate OTII CKO (*Zfx*^flox/y^ CD4-Cre^+^ OTII) mice, female *Zfx*^flox/flox^ CD4-Cre^+^ mice were crossed with male OTII mice (36). A similar strategy was employed to generate OTI *Zfx*^flox/y^ CD4-Cre^+^ mice (37). Controls were *Zfx* wild-type Cre^+^ mice as well as Cre^−^ *Zfx*^flox/y^ littermates; these mice showed similar results in all experiments. RAG1^−/−^ or RAG2^−/−^ mice were obtained from Jackson Laboratories (38). All animal experiments were performed according to the investigator’s protocol approved by the Institutional Animal Care and Use Committee. PCR of null, flox, and wild-type *Zfx* alleles was performed as described previously (28). The PCR primers used for genotyping [described in Ref. (28) in Figure S1 in Supplementary Material] are: primer A, ATTGCATGGGCAGCTGCTTAC; primer B, AGACCACTGGAAATGCCTAGC; primer C, CTTAGCACCCGTTCACTGGTC.

For all experiments in which tamoxifen was utilized to induce Cre expression in a Rosa26-CreER mouse, 50 mg tamoxifen was suspended in 1 mL sunflower seed oil; 100 μL of this suspension was administered on three consecutive days by gastric gavage to induce Cre expression.

### T Cell Analysis

For all flow cytometry experiments, single cell suspensions were generated from thymus, spleen, or lymph nodes as indicated, and stained with the following fluorochrome-conjugated antibodies from eBioscience: CD3, CD4, CD8, CD62L, and bromodeoxyuridine (BrdU). Annexin-V staining was performed according to the protocol provided by Trevigen, Inc. Samples were acquired using an LSRII flow cytometer or sorted on a FACSAria cell sorter (BD Immunocytometry Systems) and analyzed using FlowJo software (TreeStar Inc.).

### BrdU Uptake

For BrdU pulse-chase experiments, mice were intraperitoneally injected with 1 mg BrdU at the start of the pulse phase andadministered 0.8 mg/mL BrdU in drinking water for the duration of the pulse phase. After completion of the chase phase, single-cell suspensions were generated from the spleen and stained with an antibody against BrdU according to the BD PharMingen BrdU Flow Kit protocol.

### Homeostatic Proliferation Assay

Splenic T cells were enriched by negative selection against Ter119, CD11b, CD11c, B220, Gr1, and Dx5-expressing cells using magnetic-activated cell sorting (MACS; Miltenyi Biotec, Auburn, CA, USA). Subsequently, naïve cells were enriched by positive selection for CD62L expression. For homeostatic proliferation experiments, whole splenocytes or MACS-sorted naïve CD62L^+^ T cells from spleen and lymph node were stained with carboxyfluorescein succinimidyl ester (CFSE). 2–6 × 10^6^ CFSE-labeled cells were transferred intravenously into Rag1^−/−^ or Rag2^−/−^ mice by subocular injection. Control mice were Cre^−^ Zfxf^lox/y^ littermates. Lymph nodes were analyzed for CFSE dilution as noted.

### *In Vivo* Antigenic Stimulation Experiments

For *in vivo* proliferation in response to *Listeria monocytogenes*-OVA, the transgenic LM were grown at 37° to an approximate OD of 0.1, and approximately 5,000 LM-OVA were intravenously injected per mouse. Proliferation of OVA-specific T cells was monitored with a class I OVA-tetramer (Beckman-Coulter, Brea, CA, USA). For experiments involving OT-I transfer, 500–5,000 CD4-Cre OT-I or *Zfx*^(flox/y)^ CD4-Cre OT-I cells were intravenously injected into a wild-type host, followed 1 day later by LM-OVA infection. For recall response, 500,000 LM-OVA were injected to the immunized mouse.

For *in vivo* stimulation of OTII cells, two million CFSE-labeled OT-II cells were intravenously injected into recipient mice; the following day, 100 µg OVA peptide mixed 1:1 with Complete Freund’s Adjuvant was injected intraperitoneally; proliferation in the spleen and lymph nodes was assessed 3 days later.

For *in vitro* IL-15 assays, splenic T cells were MACS purified by negative selection and cultured 500,000 cells/well of a 96-well plate with 50 ng/mL IL-15.

### Immunizations and Enzyme-Linked Immunosorbent Assays

8- to 12-week-old mice were immunized by intraperitoneal injection of 50 µg NP(28)-keyhole limpet hemocyanin (KLH) mixed with alum (Biosearch Technologies, Novato, CA, USA). Mice were boosted with NP(28)-KLH in PBS on day 42 post-immunization. Serum antibody titers were determined by enzyme-linked immunosorbent assay. Anti-Ig isotype antibodies were used as capture reagents for serum antibodies.

### Protein and Gene Expression Analysis

For Western blot experiments, CD62L^+^ naïve splenic T cells from control and CKO mice were MACS-isolated, and subsequently stimulated *in vitro* with 3 µg/mL αCD3 and 2 µg/mL αCD28 in a 96-well plate with DMEM. After 2 or 12 h of stimulation, the cells were harvested and lysed, after which, they were probed with antibodies to pJNK, JNK, pERK, ERK, pEIF2α, EIF2α, and Cdkn1a (Cell Signaling).

Quantitative real-time polymerase chain reaction was performed as previously described (28, 39).

### *In Vitro* T Cell Stimulation

Naïve CD4+CD62L+ splenocytes were sorted from spleens of control or Zfx CKO, aged 6–8 weeks. Cell stimulation involved addition of the sorted cells to plate-bound aCD3 (3 mg/mL) and aCD28 (2 mg/mL); these cells were collected after 12 h and RNA was extracted for RNA-Seq.

### RNA-Seq

Control and CKO splenic T cells were FACS purified based on expression of CD4 or CD8, and RNA was subsequently isolated with Qiagen RNeasy kit. 10 μg of total RNA was then rRNA-depleted, the product of which was then treated according to Applied Biossystems SOLiD Whole Transcriptome Sequencing protocol.

Sequencing reads were mapped to the mouse reference genome (GRCm38.85/mm10) using the STAR anligner (v2.5.0c) (39). Alignments were guided by a Gene Transfer File (Ensembl GTF GRCm38.85). The mean read insert sizes and their SDs were calculated using Picard tools (v.1.126).^1^ The read count tables were generated using HTSeq (v0.6.0) (40), normalized based on their library size factors using DESeq (v3.7) (41), and differential expression analysis was performed. The Read Per Million normalized BigWig files were generated using BEDTools (v2.17.0) (42) and bedGraphToBigWig tool (v4), and downstream statistical analyses and generating plots were performed in R environment (v3.1.1).^2^ Raw RNA-Seq data have been deposited in the Gene Expression Omnibus database under the accession number GSE114785.

## Results

### Zfx Deletion Causes a Progressive Loss of Peripheral T Cells

To knock out *Zfx* in peripheral T cells, we utilized a CD4-Cre mouse to excise a floxed *Zfx* allele (28, 34). In this model, the *Zfx* allele is excised in all T cells at the double-positive stage of T cell development (34). The resulting CKO mice displayed total thymocyte numbers and distribution of thymocyte subsets that were comparable to control mice, revealing that CD4-Cre-driven excision of *Zfx* in these mice does not perturb T cell development (Figures S1A,B in Supplementary Material).

**Figure 1:**
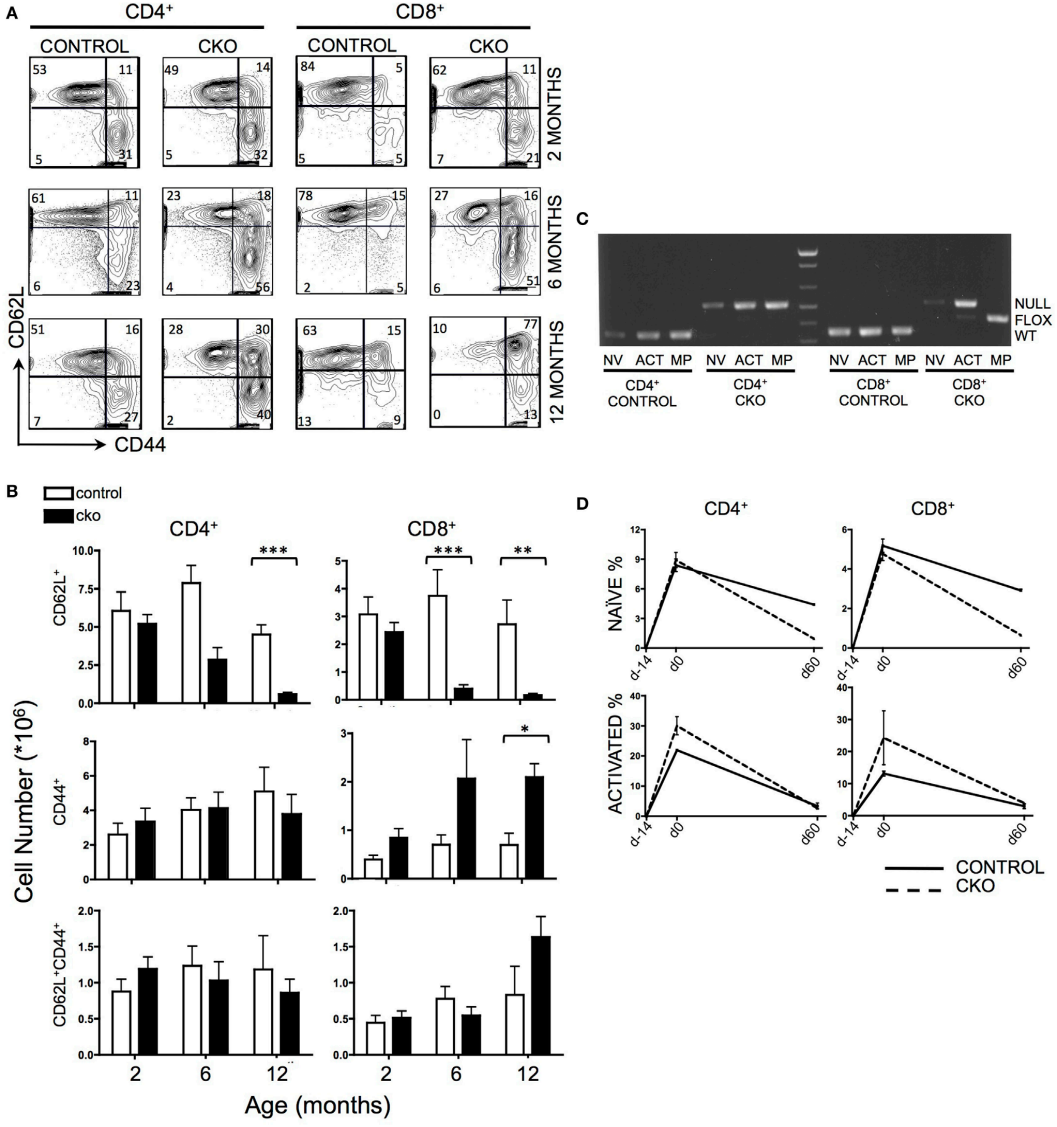
Zfx deletion causes a time-dependent loss of naïve peripheral T cells, and an accumulation of Zfx “non-deleters.” **(A)** Splenic T cell populations in control and conditional knockout (CKO) mice over time. Shown are FACS profiles of splenic T cell CD62L and CD44 expression from mice of the indicated ages. Numbers represent percentage of cells in each gate. Plots are representative of over five independent experiments. **(B)** Summary of T cell population numbers in control and CKO mice over time. The plots show absolute number ± SEM of each indicated splenic T cell population in control and CKO mice at 2 months (*n* = 8–10), 6 months (*n* = 5–7), and 12 months (*n* = 2–3) of age. Statistically significant differences are indicated as follows: *** *P* < 0.001; **P < 0.01; *P < 0.05. **(C)** PCR of Zfx allele in splenic T cells of 10-month-old mice. Shown is a PCR that detects the presence of a deleted (top band) or floxed non-deleted (bottom band) Zfx allele from FACS-sorted T cell populations. NV = naïve (CD62L^+^), ACT = activated (CD44^+^), MP = memory phenotype (CD62L^+^CD44^+^). **(D)** Formation and loss of peripheral T cells over time. Control and CKO mice (less than 3 months of age) were injected intraperitoneally with bromodeoxyuridine (BrdU) and then administered BrdU in their drinking water for 14 days, followed by removal of BrdU for 60 days. Shown are the percentages of CD62L^+^ naïve and CD44^+^ activated BrdU^+^ T lymphocytes in the spleen. The values represent percentage of BrdU^+^ cells ±SEM for two mice at each timepoint. Plots are representative of three independent experiments.

The peripheral T cell compartment consists of quiescent “naïve” cells that are characterized by surface expression of CD62L, “activated” cells that express CD44 in response to external stimulating signals [such as antigen or cytokines released in response to lymphopenia (43)], and “memory phenotype” cells that have completed homeostatic proliferation and express both CD62L and CD44. *Zfx* CKO mice displayed an age-dependent loss of CD62L^+^ naïve T cells in both the CD4^+^ and CD8^+^ T cell populations (Figures 1A,B). At 2 months of age, the T cell populations in control and CKO mice were similar. As the CKO mice age beyond 6 months, they experienced a progressive loss of CD62L^+^ cells, and in the CD8^+^ population, an increase in CD44^+^ activated cells (Figure 1B). At 12 months of age, there was a distinct population of CD8^+^CD62L^+^CD44^+^ cells in the CKO mice, which represent “memory phenotype” cells that have undergone homeostatic proliferation in response to lymphopenia (7, 8). PCR of FACS-sorted T cells revealed that this population in the CKO mice contained exclusively *Zfx*-expressing cells that had escaped deletion of the *Zfx* allele (Figure 1C). CD4-Cre excises a floxed allele with greater than 99% efficiency (44); therefore, the rare group of <1% of cells that escaped *Zfx* deletion initiated homeostatic proliferation in response to lymphopenia secondary to loss of *Zfx*-deficient T cells. This does not occur in CD4+ cells because of continued Cre expression by the CD4 promoter.

To distinguish whether the loss of CD62L^+^ *Zfx*-deficient naïve T cells was caused by impaired formation of new T cells or loss of existing T cells, mice were administered a BrdU “pulse” for 14 days, followed by a 60-day “chase” period. During the pulse, CKO cells had the same rate of BrdU incorporation as controls, suggesting that CKO cells are formed at a similar rate as controls (Figure 1D). However, the loss of BrdU^+^ cells during the chase phase was significantly greater in the CKO mice, revealing an inability of the *Zfx*-deficient T cells to persist over time. In addition, there was an increase in the rate of formation of CD44^+^ activated cells in the CKO mouse (as described previously), which also died at an increased rate as compared to *Zfx*-expressing cells (Figure 1D).

### Defective Homeostatic and Antigen-Induced Proliferation of Zfx-Deficient T Cells

**Figure 2:**
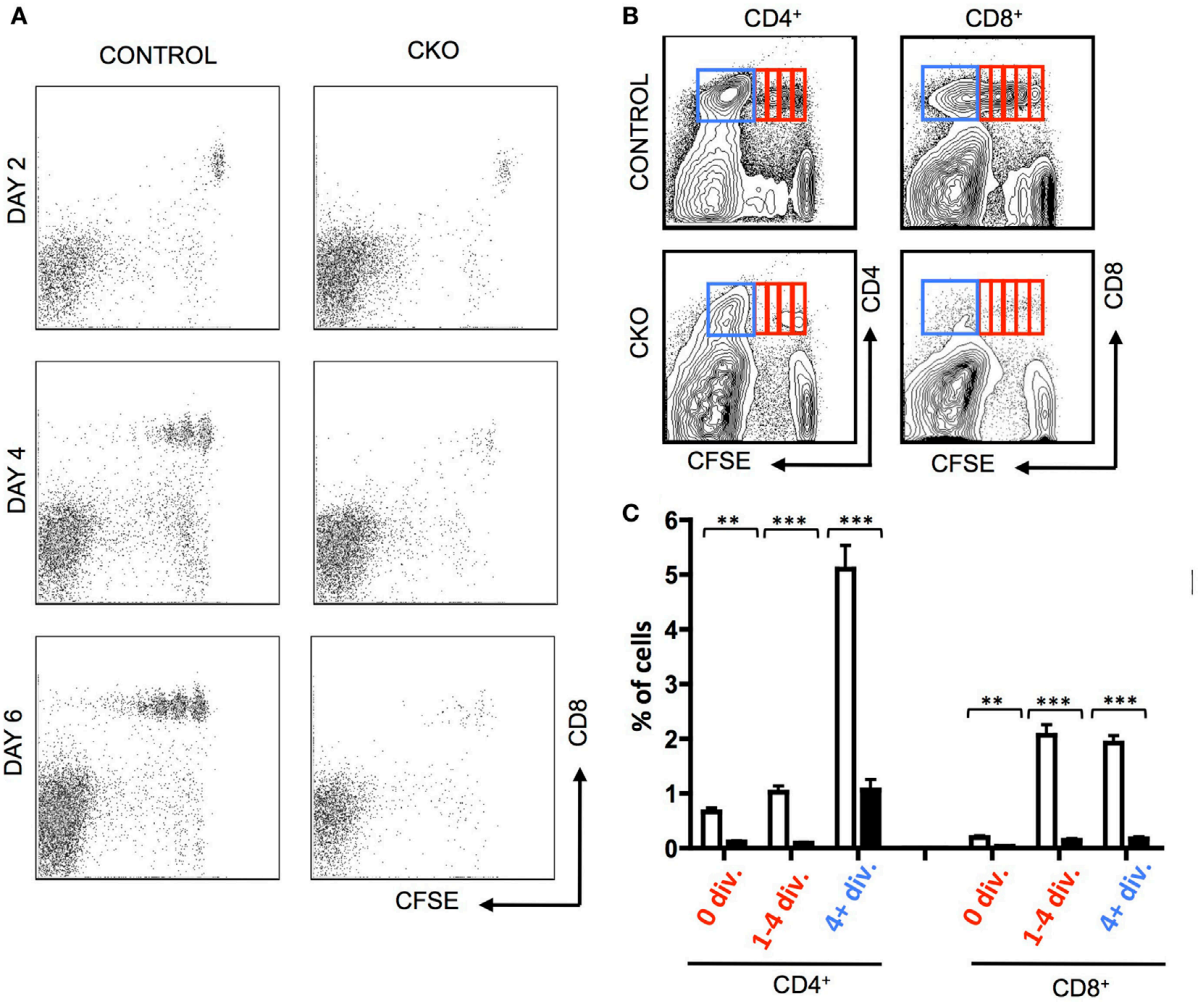
Zfx-deficient T cells fail to undergo acute homeostatic proliferation. **(A)** Expansion of T cells in a lymphopenic environment. Shown is FACS plots demonstrating carboxyfluorescein succinimidyl ester (CFSE) dilution in CD8^+^ control and conditional knockout T cells injected into RAG1^−/−^ mice and isolated from the lymph nodes at the indicated days. **(B)** Expansion of transferred T cells 7 days after transfer into RAG1^−/−^ mice. Shown is FACS plots demonstrating CFSE dilution in T cells within the lymph nodes 7 days after transfer. Data are representative of two independent experiments. **(C)** Quantification of T cell expansion 7 days after transfer into RAG1^−/−^ mice. Shown is the fraction of lymph node CD8^+^ cells that have divided 0, 1–4, or 4+ times in RAG1^−/−^ mice that received control (*n* = 5) or CKO (*n* = 3) splenocytes. Numbers represent percentage ± SEM. ***P < 0.001; **P < 0.01; *P < 0.05.

To directly examine the capacity of CKO cells for homeostatic proliferation, these cells were isolated from secondary lymphoid organs and transferred into lymphopenic Rag1^−/−^ mice. Importantly, the CKO cells were not deficient in short-term survival or homing to secondary lymphoid organs, as they were detectable in lymph nodes at day 2 (Figure 2A). By days 4–6, the CKO cells showed a striking defect in expansion when compared to control cells (Figures 2A–C). This could be caused by a defect in responding to external cues of homeostatic proliferation such as IL-7, a cell survival defect, or to an intrinsic inability to execute cell expansion. The CKO cells were capable of expanding when cultured *in vitro* with IL-7, which is a critical cytokine in driving homeostatic proliferation (Figure S2A in Supplementary Material (45)). These cells did not have an intrinsic short-term survival defect, as the CKO cells exhibited rates of apoptosis comparable to control cells when cultured *in vitro* (Figure S2B in Supplementary Material). Thus, the inability of the *Zfx*-deficient T cells to proliferate in a lymphopenic environment suggests that these cells have a general cell expansion abnormality. Of note, the CKO mice were on mixed B6/129 background, whereas the recipient RAG KO mice were pure B6. While GVHD is formally possible in this case, it is extremely unlikely because B6 and 129 share the same MHC haplotype (H2-Kb). Thus, any reactivity would only be possible against minor histocompatibility antigens and, therefore, would be very limited and/or take a long time to develop, if occurring at all. Finally, the control cells derived from 129/B6 littermates did not display the same defect as the CKO cells.

**Figure 3:**
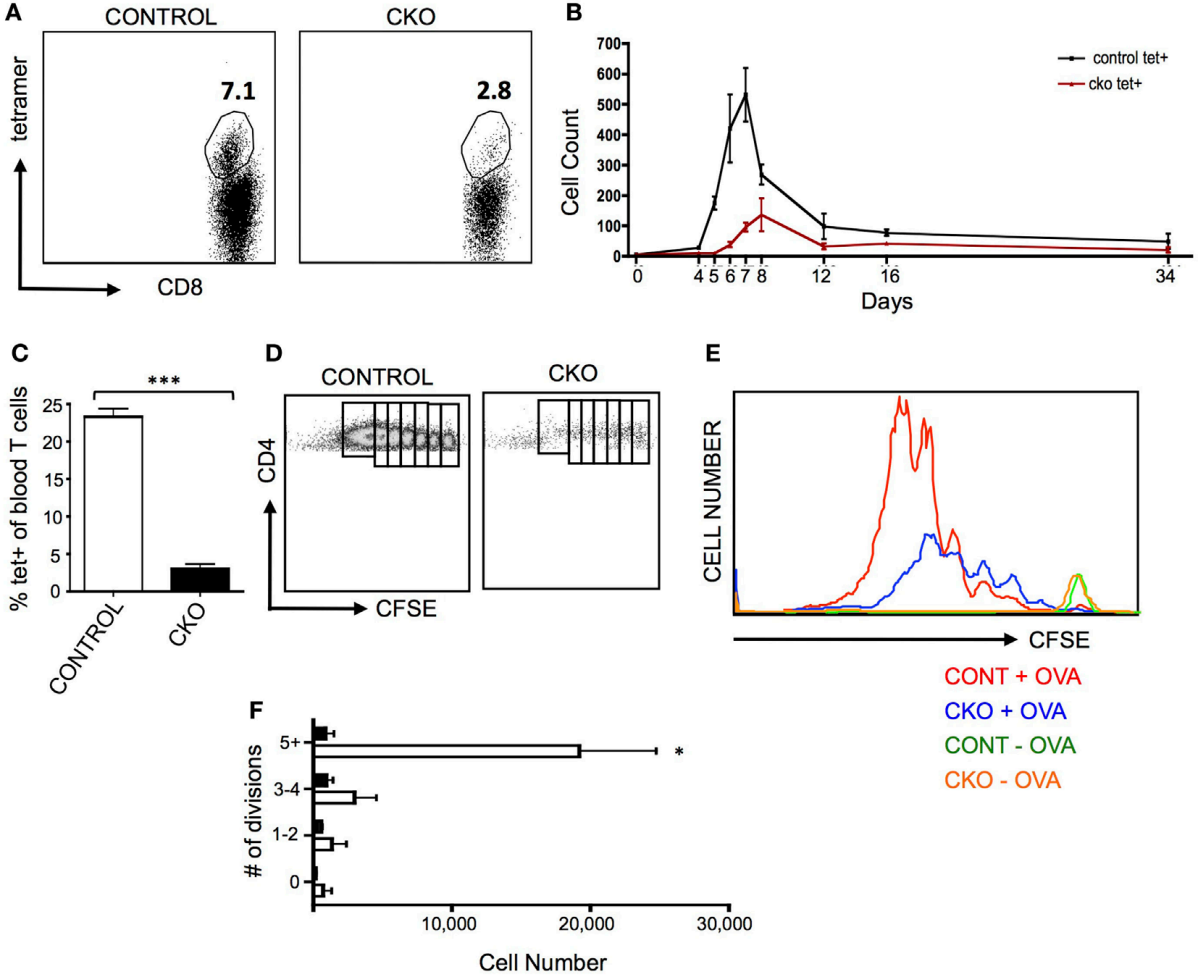
Zfx-deficient T cells fail to proliferate in response to antigen stimulation *in vivo*. **(A)** Percentage of antigen-specific CD8^+^ cells in the peripheral blood after infection with LM-OVA. Control and conditional knockout (CKO) mice were intravenously injected with approximately 5,000 LM-OVA and tetramer^+^ cells were tracked in the blood. Shown is a FACS plot of CD8^+^ cells that are tetramer^+^ in the blood at d7 postinfection. Number represents percentage of tetramer^+^ cells in the CD8^+^ cell population. **(B)** Expansion of antigen-specific CD8^+^ cells after injection of LM-OVA. Shown is the absolute number of tetramer^+^ cells in the blood over the course of LM-OVA infection. Values represent number of tetramer^+^ cells ±SEM per 100,000 live white blood cells from control (*n* = 3, black line) and CKO (*n* = 3, red line) mice. Representative of more than five experiments. **(C)** Proliferation of transferred transgenic OT-I cells 5 days after exposure to LM-OVA. 500 control or CKO OT-I cells were transferred into lymhoreplete syngeneic recipients and 1 day later infected with LM-OVA. Shown is the percentage of tetramer^+^ cells ±SEM in the blood at day 5. ***P < 0.001. **(D)** Expansion of transgenic OT-II cells. 5 × 10^6^ carboxyfluorescein succinimidyl ester (CFSE)-labeled control and CKO cells were intravenously injected into syngeneic lymphoreplete recipients, and 1 day later, the mice were injected intraperitoneally with complete Freund’s adjuvant with ovalbumin (CFA-OVA). Shown is CFSE dilution at day 3 post CFA-OVA administration. Data are representative of three independent experiments. **(E)** Quantification of expanded cell populations. Shown is the dilution of CFSE versus the absolute number of cells at each stage of CFSE dilution, for control + CFA-OVA (red), CKO + CFA-OVA (blue), control without CFA-OVA (orange), and CKO without CFA-OVA (green). **(F)** Quantification of splenic T cells at each stage of cell division 3 days after CFA-OVA administration. Shown is absolute number of OTII T cells in the spleen ±SEM.

A common pathogenic model for the study of antigen-induced CD8^+^ T cell proliferation is infection with *Listeria monocytogenes* that expresses the antigen ovalbumin (LM-OVA), which allows for tracking of antigen-specific cells in the blood and lymphoid organs by staining with a T cell receptor-specific tetramer (46). When CKO mice were infected with LM-OVA, the proliferative expansion was greatly diminished, both in magnitude as well as time needed to obtain the peak number of T cells (Figures 3A,B). This proliferative defect was cell intrinsic, as *Zfx*-deficient CD8^+^ OT-I transgenic cells also had a deficient proliferative response upon transfer into a wild-type mouse that was then infected with LM-OVA (Figure 3C).

To determine the capacity of *Zfx*-deficient CD4^+^ T cells for antigen-induced proliferation, OTII cells were transferred to wild-type recipients that were then injected intraperitoneally with complete Freund’s adjuvant with ovalbumin (CFA-OVA). Three days after CFA-OVA injection, the CKO cells displayed greatly diminished expansion compared to controls (Figures 3D–F). Importantly, in mice that were never treated with CFA-OVA, CKO cells were found in the spleen in comparable numbers to control cells, supporting the conclusion that the *Zfx*-deficient T cells do not have a migration defect (Figure 3E, green and orange plots). Thus, the inability of CKO CD4^+^ and CD8^+^ cells to proliferate in response to antigen suggests that these cells have a pervasive inability to initiate a program of expansion in response to external stimuli. Surprisingly, antibody production in response to NP-KLH immunization was not affected in the CKO mice, revealing that these mice did not display a defect in the capacity to stimulate B cell antibody production (Figure S3A in Supplementary Material).

### Abnormal Memory T Cell Formation and Response to Secondary Antigen Challenge in Zfx-Deficient T Cells

**Figure 4:**
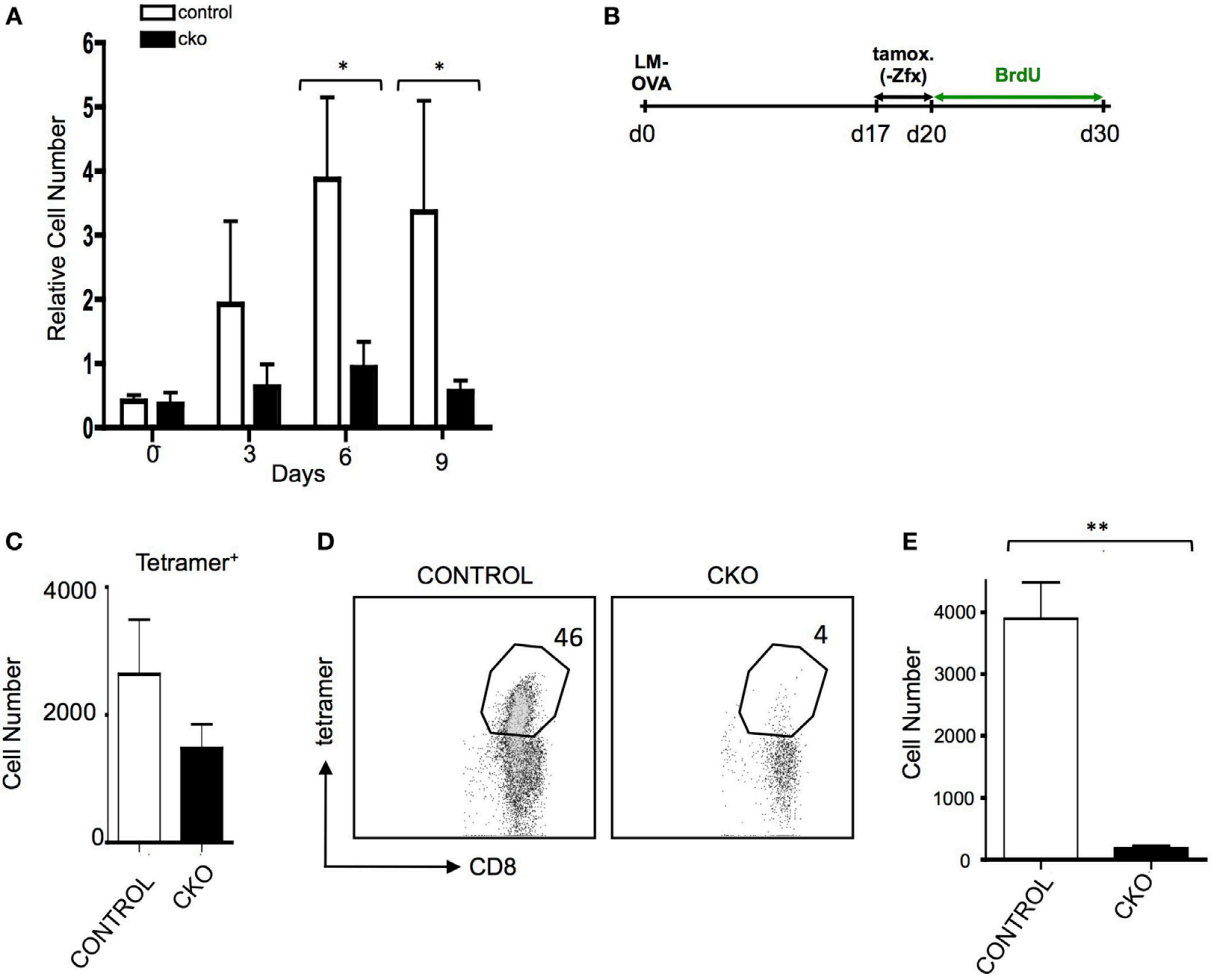
Zfx-deficient T cells have a defect in cell turnover and an impairment in recall responses. **(A)** Memory phenotype (MP) cell expansion in response to IL-15. MACS purified splenic T cells were cultured with 50 ng/mL Il-15. Shown is the relative expansion of control or conditional knockout (CKO) CD8^+^CD44^+^CD62L^+^ MP cells. Numbers represent the average increase in cell number ± SEM relative to day 0 (*n* = 3). *P < 0.05. **(B)** Experimental layout for figures **(C–E)**. Control and CKO Rosa26-CreER mice were infected with LM-OVA, and on days 17–19, postinfection administered tamoxifen in order to induce Zfx deletion. On day 20, mice were injected intraperitoneally with bromodeoxyuridine (BrdU) and then administered BrdU in their drinking water for 10 days. Tetramer^+^ and BrdU + cells were tracked as noted in the figures. **(C)** Formation of memory T cells. Mice were treated according to Figure 4. Shown is the average ± SEM of tetramer^+^ cells per 1 million splenocytes on day 30, 10 days after Zfx deletion (*n* = 2, graph is representative of two independent experiments). **(D)** Proliferation of memory T cells in response to secondary exposure to antigen. Control and CKO Rosa26-CreER mice were infected with LM-OVA and on days 48–50 administered tamoxifen; on day 52, the mice reinfected with 100-fold more LM-OVA. Shown is the percentage of CD8^+^ cells in the blood that are tetramer^+^ 4 days after secondary challenge. **(E)** Quantification of tetramer^+^ memory T cells in the blood 4 days after re-challenge with LM-OVA. Shown is the absolute number ± SEM of tetramer^+^ cells per 100,000 blood cells in control and CKO Rosa26-CreER mice, treated as in **(D)**. **P < 0.01.

The inability of CKO cells to properly form effector T cells upon antigen exposure limits the ability to adequately study the formation of memory T cells after effector cell contraction. Administration of exogenous IL-15 causes selective growth of CD8^+^ memory phenotype (MP) cells (47), which have many phenotypic and gene expression patterns similar to antigen-experienced memory cells (7). Indeed, treatment of CKO T cells with IL-15 revealed a diminished capacity for expansion (Figure 4A). To study memory T cells independent of effector cell expansion *in vivo*, Rosa26-CreER mice were utilized to delete *Zfx* after effector cell contraction (35). To do this, CKO *Zfx*^(flox/y)^ Rosa26-CreER and control Rosa26-CreER mice were infected with LM-OVA to induce an effector T cell response; on days 17–20, tamoxifen was administered to induce deletion of *Zfx* after the contraction of effector cells. On day 21, the mice were injected intraperitoneally with BrdU and administered BrdU in their drinking water for the next 10 days (Figure 4B). By day 30, the *Zfx*-deficient memory T cell pool was decreased compared to control cells (Figure 4C). To examine *Zfx*-deficient memory T cell expansion in response to antigen re-exposure, Rosa 26-CreER mice were again infected with LM-OVA, and on days 47–50 fed tamoxifen to induce deletion of *Zfx*. On day 51, these mice were re-challenged with LM-OVA to induce memory T cell expansion. Four days after re-challenge, the *Zfx*-deficient memory T cells were diminished more than 10-fold in their capacity to expand and mount an effective memory T cell response (Figures 4D,E).

### Zfx is Essential in the Development of iNKT Cells

**Figure 5:**
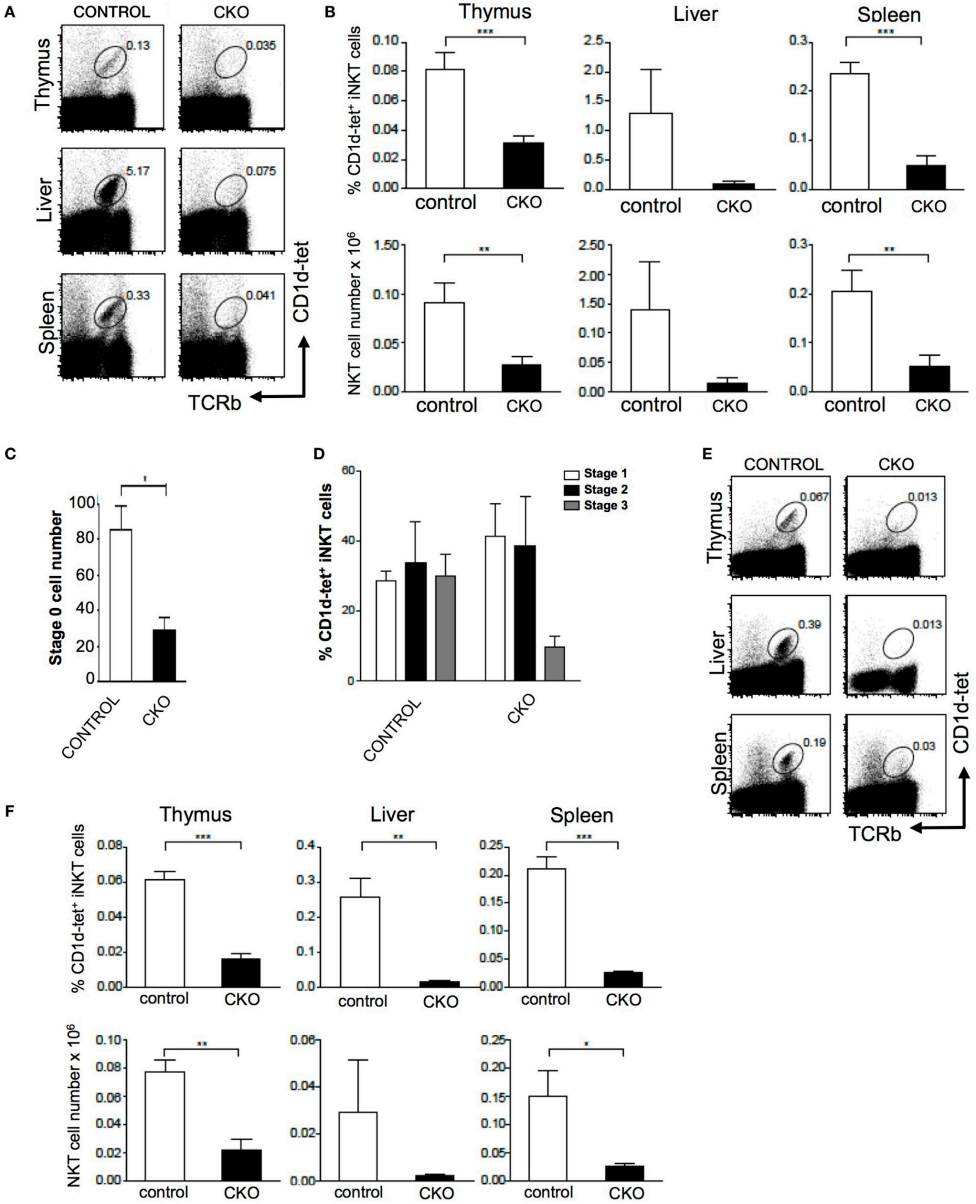
Zfx is required for homeostasis of invariant natural killer T cell (iNKT) cells. **(A)** Zfx deficiency in double-positive thymocytes inhibits iNKT cell maintenance. Shown is the percentage of TCRβ^+^CD1d-tetramer^+^ iNKT cells in the thymus, liver, and spleen of control and conditional knockout (CKO) (CD4-Cre Zfx^flox/y^) mice. Data are representative of 1–3 mice over two different experiments. **(B)** Quantification of iNKT cells in control and CKO mice. Top row shows the percentage of TCRβ^+^CD1d-tetramer^+^ iNKT cells, and the bottom row contains the number (×10^6^) of iNKT cells in the thymus, liver, and spleen. **P < 0.005 and ***P < 0.0005. **(C)** Zfx deficiency causes decreased formation of stage 0 iNKT cells. Shown is the number of CD1d-tetramer^+^CD24^+^ stage 0 cells after enrichment for CD1d-tetramer^+^ cells by magnetic-activated cell sorting. Shown is quantification of the number ± SEM of stage 0 iNKT cells. *P < 0.05. **(D)** Zfx deficiency impairs iNKT cell development. Shown is quantification of the percentage of cells in stage 1, 2, and 3 of iNKT cell development based on expression of CD44 and NK1.1. Data are representative of 1–3 mice in two independent experiments. **(E)** iNKT cell developmental defect is intrinsic to the bone marrow compartment. Shown is expression of TCRβ and CD1d-tetramer of Zfx-deficient iNKT cells in the thymus, liver, and spleen after reconstitution of irradiated recipients with 1:1 mixture of wild-type and CKO bone marrow. **(F)** Quantification of iNKT cells in bone marrow chimeric mice. The top row shows the percentage of Zfx-deficient TCRβ^+^CD1d-tetramer^+^ iNKT cells in thymus, liver, and spleen. The bottom row shows the number (×10^6^) of iNKT cells in the thymus, liver, and spleen. *P < 0.05, **P < 0.005, and ***P < 0.0005.

The *Zfx*^(flox/y)^ CD4-Cre mouse was used to assess the role of *Zfx* in iNKT cell development (11, 12). Importantly, conventional αβ T cell development was unperturbed in the *Zfx^flox^*^/y^ CD4-Cre mice (Figure S1 in Supplementary Material). Staining for CD1d tetramer and TCRβ expression revealed that *Zfx* deficiency caused a loss of iNKT cells in the thymus, liver, and spleen (Figures 5A,B). Detection of developing iNKT cells in the thymus revealed impaired maturation in the CKO beginning at stage 0, and also manifesting at stage 3 of iNKT cell development (Figures 5C,D). To determine whether this developmental defect was intrinsic to the bone marrow compartment, irradiated littermate recipient mice were reconstituted with *Zfx* CKO donor bone marrow, and the development of *Zfx*-deficient iNKT cells was analyzed. The *Zfx*^flox/y^ CD4-Cre bone marrow was unable to generate iNKT cells to comparable levels of control bone marrow (Figures 5E,F). Thus, Zfx is also required for the proper development of iNKT cells.

### Zfx Controls a Common Program of Cell Division in T Cells and HSC

**Figure 6:**
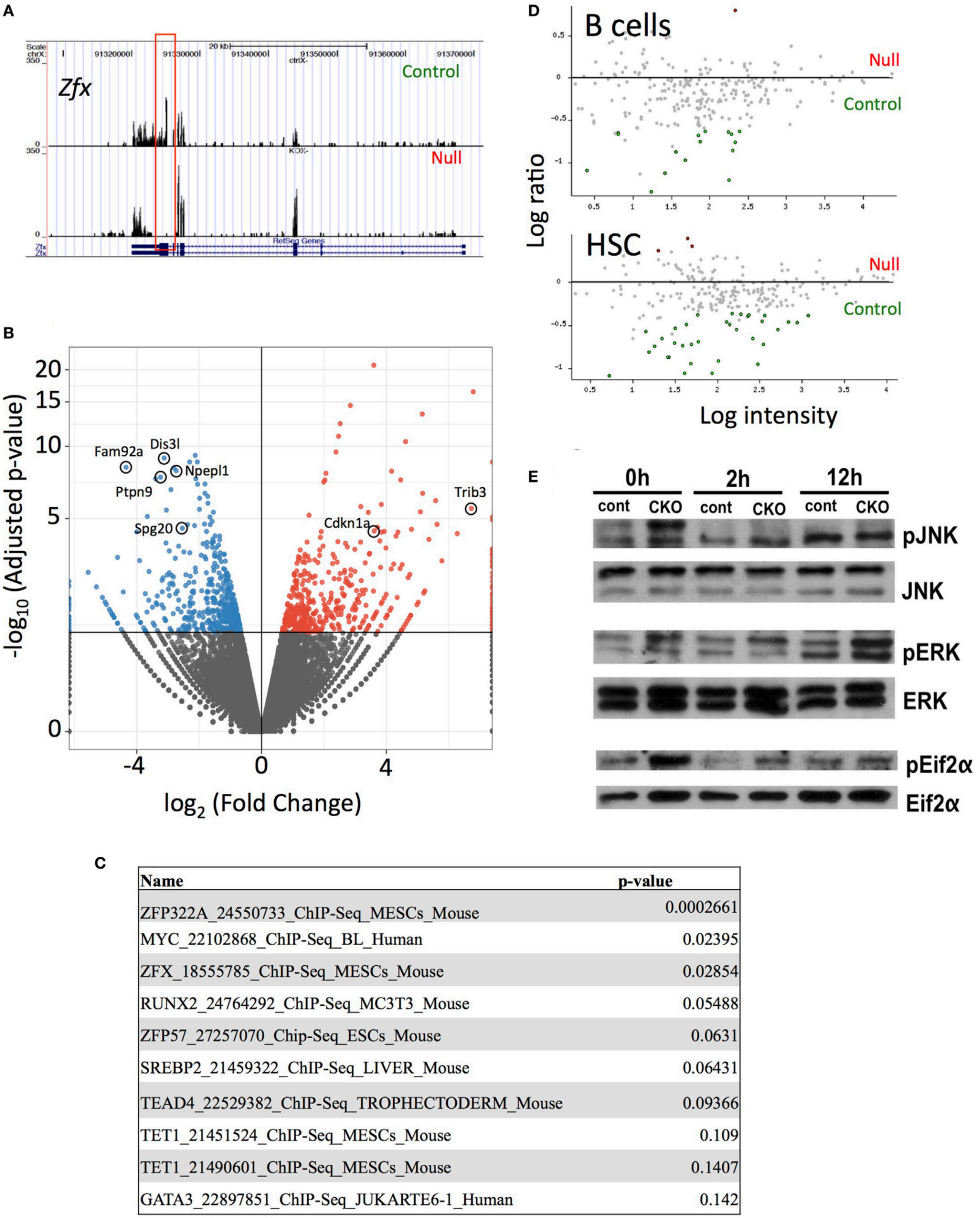
Loss of Zfx activates a transcriptional program in T cells similar to that observed in Zfx-deficient B cells and hematopoietic stem cell (HSC). **(A)** Gene expression profiles on Integrated Genome Viewer. RNA was isolated from FACS sorted splenic T cells, which was used for high-throughput RNA sequencing. Shown is a comparison sequencing result of control and conditional knockout (CKO) cells for Zfx. **(B)** Summary of the RNA-seq results. Volcano plot representation of differential expression analysis of genes in the control versus Zfx CKO T cells. Red and blue points mark the genes with significantly increased or decreased expression, respectively, in control compared to Zfx-null samples. The *x*-axis shows log_2_ fold-changes in expression and the *y*-axis the adjusted *P*-value. **(C)** ChIP-CHIP/Seq datasets showing greatest enrichment for the significantly downregulated protein-coding genes, according to ChEA 2016. List generated based on protein-coding downregulated genes with adjusted *P*-value < 0.05. Shown are the datasets and *P*-values. **(D)** Cell-specific expression of genes downregulated in T cell CKO. Each dot represents a gene that was strongly downregulated in Zfx-deficient T cells based on RNA sequencing, plotted according to upregulation (red) or downregulation (green) in gene expression microarrays performed in B cells and HSC. **(E)** Zfx-deficient T cells display activation of the unfolded protein response. Control and CKO splenic T cells were MACS isolated and stimulated with 3 µg/mL αCD3 and 2 µg/mL αCD28 for 0, 2, or 12 h *in vitro*. Shown is western blot of the indicated T cell populations.

Next-generation RNA sequencing was performed to determine the gene expression abnormalities in Zfx-deficient T cells. Previous studies, using expression microarrays and ChIP-on-CHIP, revealed a set of genes that are direct transcriptional targets of Zfx in ESC, and are significantly downregulated upon Zfx deletion in ESC, HSC, and B cells. Analysis of whole T cell populations from the spleen of young (<2 months) mice confirmed the absence of exons 10–11 in the CKO cells, as expected based on the location of the deletion (Figure 6A). In addition, *Fam92a* and *Dis3l*, two genes previously shown to be direct transcriptional targets of Zfx in mESC and downregulated in Zfx-deficient B cells, ESC, and HSC, were confirmed to be similarly decreased in the CKO T cells [Figure 6B (28)]. Furthermore, many genes downregulated in Zfx-deficient HSC were similarly decreased in the Zfx-deficient T cells, including *Ptpn9, Npepl1*, and *Spg20* [Figure 6B (28)]. A similar pattern was observed in T cells that were stimulated *in vitro* with anti-CD3 and anti-CD28 for 12 h (Figure S4 in Supplementary Material). This suggests a common genetic program controlling cellular proliferation in HSCs and lymphocytes. In addition, analysis of the most significantly downregulated protein-coding genes with the ChEA 2016 program [which searches ChIP-CHIP/Seq datasets (48)] revealed significant enrichment with the Zfx-deficient mESC ChIP-CHIP dataset [*P*-value 0.029, Figure 6C (28)]. To further explore this possible common genetic program, the most dysregulated genes in the CKO T cells were compared to previous microarrays in HSC and B cells (28, 29); this revealed that the most dysregulated genes in CKO T cells were similarly dysregulated in Zfx-deficient HSC and B cells (Figure 6D). Thus, the gene expression pattern of Zfx-deficient T cells is similar to that of Zfx-deficient HSC, suggesting that there is a common genetic pathway regulating the self-renewal of both cell types.

Previous experiments in B cells revealed an induction of the integrated stress response (ISR) upon Zfx deletion (29). Similarly, there was a significant increased expression of genes involved in the ISR in Zfx-deficient T cells, including Cdkn1a and Trib3 (Figure 6B). There was also increased phosphorylation of JNK, ERK, and Eif2α, which represents activation of the unfolded protein response, which is closely intertwined with the ISR in immune cells [Figure 6E (49–51)]. In sum, Zfx-deficient T cells show similar gene expression patterns as Zfx-deficient HSC, suggesting a common mechanism of regulation of self-renewal, and dysregulation of this common pathway leads to activation of the unfolded protein response.

## Discussion

We have shown that the zinc finger transcription factor *Zfx* is essential for the long-term maintenance of naïve and memory T cells in the periphery, as well as primary and secondary proliferative responses to antigen. Furthermore, *Zfx*-deficient T cells failed to undergo acute homeostatic proliferation in response to lymphopenia. *Zfx* was also shown to be essential for the development of iNKT cells.

Molecular control of peripheral T cell self-renewal is poorly understood, largely because the process occurs rarely in naïve T cells, and most of the cells at any one time are not cycling. Whether there is a specific molecular program for the chronic homeostatic self-renewal of T cells is unclear. In the few cases in which deletion of a gene leads to a specific loss of T cell number, it is challenging to differentiate between a defect in self-renewal as opposed to a defect in other processes such as survival.

The impaired chronic maintenance of *Zfx*-deficient naïve T cells seems to be more than a simple survival defect, because it takes approximately 6 months for the phenotype to manifest. This mimics the role of *c-Myc*, which is essential for proliferation in T cell development, but is dispensable for survival and differentiation (52). The outgrowth of Zfx non-deleters that parallels the loss of *Zfx*-deficient peripheral T cells is a striking phenotype. To the best of our knowledge, the outgrowth of rare non-deleting cells has only been described in a single instance, when CD4-Cre was utilized to drive expression of diphtheria toxin A in order to kill all recombined cells (53), allowing for homeostatic proliferation of the rare cells that failed to recombine.

The defects observed in *Zfx*-deficient T cells resembles the self-renewal defect in *Zfx*-deficient HSC. Our RNA-Seq results suggest that there is a common mechanism of cell division shared specifically by HSC and lymphocytes. Deletion of *Zfx* affects the homeostasis of HSC, T cells, and B cells, without having an apparent effect on any other tissues or organs. This was most strikingly revealed when *Zfx*-deficient ESC contributed to all tissues of chimeric mice except for the bone marrow and thymus. In addition, induced *Zfx* deletion in Mx1-Cre mice revealed defects in HSC and lymphoid progenitors, but normal myeloid and erythroid compartments (28). We further narrowed the hematopoietic defect by studying the production of mature cells following conditional deletion of *Zfx* in a variety of hematopoietic precursor cells. Indeed, deletion of *Zfx* specifically in dendritic cells caused no apparent defect in their function (data not shown). It is, therefore, likely that *Zfx* uniquely regulates the proliferation of both HSC and lymphocytes by a similar mechanism. In this study, rescue of the phenotypic abnormalities by Zfx overexpression was not feasible, because we and others have observed that overexpression of Zfx cDNA from a heterologous promoter (e.g., from a plasmid or retrovirus) is invariably toxic to cells. Thus far, heterologous expression of Zfx has been possible only by inserting and entire Zfx genomic locus in a BAC (28, 54) and, therefore, could not be used for genetic rescue *in vivo*. Importantly, such heterologous expression was able to rescue the phenotype of Zfx-deficient ES cells (28), confirming the specificity of Zfx deletion.

There are a few cases in which a T cell subtype displayed properties very similar to a HSC. For example, some chronic virus-specific memory T cells consistently survive multiple rounds of chemotherapy because they are quiescent and express multidrug efflux proteins, much like HSC (55). Similarly, development of graft-versus-host disease in mice seems to coincide with development of stem cell-like naïve T cells that are host tissue-specific and exhibit self-renewal as well as the ability to produce all effector and memory subsets (3).

There are very few genes that have been described to play a similar role exclusively in stem cells and lymphocytes, as we have observed for *Zfx*. There are a number of genes that are important for both HSC and T cell homeostasis, but in many cases, these genes are also important in other tissues that do not display properties of self-renewal and multipotency. For example, *Bmi-1* and *c-Myb* are important in controlling proliferation or survival in HSC and T cells (56, 57), but they are also serve essential functions in fibroblast proliferation [for *Bmi-1* (58)], and smooth muscle differentiation [for *c-Myb* (59)]. In addition, some genes are important for T cell and HSC homeostasis, but seem to play vastly different roles in the two different cell types. This is exemplified by *Runx1* and *GATA3*, which are important in HSC maintenance (60, 61), while also being essential in T cells for differentiation into various T helper subsets (62). Therefore, while some genes are similarly essential for HSC and T cell function, in most cases, these genes do not control the same function in HSC and T cells, and often, these genes have roles in other tissues as well. In addition, the role of these factors in T cell homeostasis is often specific to one or two processes, rather than permeating across all homeostatic decisions encountered by a T cell.

The genes previously described as direct transcriptional targets of *Zfx* in ESC and HSC are downregulated upon *Zfx* deletion in T cells. It is, therefore, tempting to speculate that *Zfx* mediates a specific transcriptional program that is uniquely important in HSC and lymphocytes. There is no information connecting these genes, including *Fam92a, Dis3l*, and *Ptpn9*, to a common cellular pathway. Not much is known about the role of *Fam92a* or *Ptpn9* in the hematopoietic system. *Dis3l* was recently described to regulate levels of Y RNA, which are poorly understood small RNAs that are important in DNA replication (63). Future work will be directed at elucidating the mechanism by which these genes control an important program of self-renewal in the hematopoietic system.

Multiple recent studies have revealed that *Zfx* is important in the progression of various cancers, including renal cell carcinoma, leukemia, and glioblastoma (31–33). *Zfx* is upregulated a diverse array of human cancers, and it is known that knockout of *Zfx* in mice prevents development as well as maintenance of leukemia (33). Better understanding of the molecular mechanisms of *Zfx* in hematopoietic cells has the potential to also improve our models of cancer pathogenesis.

*Zfx* is relevant to a variety of clinical phenomena. As stressed above, its role in the self-renewal of HSC as well as T lymphocyte cell division suggests that there is a commonality in these two processes beyond the simple fact that both cells divide. Once we understand why *Zfx* is essential in HSC, there is potential to extrapolate this information to improve HSC-related clinical therapies, such as bone marrow transplants. Similarly, understanding how *Zfx* controls T cell homeostasis has the potential to improve treatments ranging from immunizations to HIV therapies. This might also lead to a clearer image of cancer development and the exact cellular processes that must be intact for cancerous transformation.

## Ethics Statement

All animal experiments were performed according to the investigator’s protocol approved by the Institutional Animal Care and Use Committee.

## Author Contributions

MS-R and BR designed the experiments. MS-R and TA performed all experiments with the exception of the iNK data, which was generated by LD and AG. AK-J and AT contributed to the analysis of RNA-Seq data. MS-R wrote the manuscript with input from BR.

## Conflict of Interest Statement

The authors declare that the research was conducted in the absence of any commercial or financial relationships that could be construed as a potential conflict of interest.

## Footnotes

^1^http://broadinstitute.github.io/picard/ (Accessed: August 5, 2018).

^2^http://www.r-project.org/ (Accessed: August 5, 2018).

## Supplementary Material

Figure S1 Zfx(fl/y) CD4-Cre mice progress normally through the stages of T cell development. **(A)** Developing T cell populations in the thymus. Shown are FACS profiles of thymocytes in control and conditional knockout mice; numbers represent the percentage of each population and are representative of over five experiments. **(B)** Distribution of thymocyte populations. Shown are absolute numbers ± SEM of various thymocyte populations based on expression of CD4 and CD8 as detected by flow cytometry; DP, double positive. Data are representative of two to five mice. **(C)** Distribution of thymocyte populations. Shown are absolute numbers ± SEM of various thymocyte populations based on expression of CD4 and CD8 as detected by flow cytometry. DN, double negative. Data are representative of two to five mice.

Figure S2 Zfx-deficient T cells have normal response to IL-7 and display normal levels of apoptosis *in vitro*. **(A)** *In vitro* expansion of T cells in response to IL-7. CD62L^+^ naïve T cells were isolated and maintained in 1 ng/mL IL-7 for 6 days. Shown is the fold expansion of control and conditional knockout (CKO) cells on days 3 and 6, for CD4^+^ (left) and CD8^+^ cells (right). Data are representative of more than three independent experiments. **(B)** *In vitro* apoptosis. Control and CKO splenic T cells were isolated and maintained in culture for 6 and 24 h, after which, they were stained for Annexin-V and 7-AAD. Numbers represent the percentage of early (Ann-V^+^7-AAD^−^) and late (Ann-V^+^7-AAD^+^) apoptotic cells; data are representative of two independent experiments.

Figure S3 Zfx deficiency has minimal effect on stimulation of B cell antibody production. Zfx-deficient T cells can drive B cell antibody production *in vivo*. Control (open triangles) and conditional knockout (CKO) (CD4-Cre Zfx^flox/y^; closed circles) mice were immunized with NP-keyhole limpet hemocyanin (KLH) and antibody titers were measured by ELISA every week post-immunization, for 7 weeks. Mice were boosted with a second dose of NP-KLH on day 42. Shown are relative antibody titers from five control and five CKO mice.

Figure S4 Zfx-deficient T cells simulated *in vitro* display similar expression defects as unstimulated T cells as well as hematopoietic stem cells. Summary of the RNA-seq results. Volcano plot representation of differential expression analysis of genes in the control versus Zfx conditional knockout T cells. Red and blue points mark the genes with significantly increased or decreased expression, respectively, in control compared to Zfx-null samples. The *x*-axis shows log_2_ fold-changes in expression and the *y*-axis the adjusted *P*-value.
