# Supplementary figures and images for: The Transcription Factor Zfx Regulates Peripheral T Cell Self-Renewal and Proliferation
Source: Front Immunol. 2018 Jul 4;9:1482. doi: 10.3389/fimmu.2018.01482 (PMC6039547; doi:10.3389/fimmu.2018.01482)

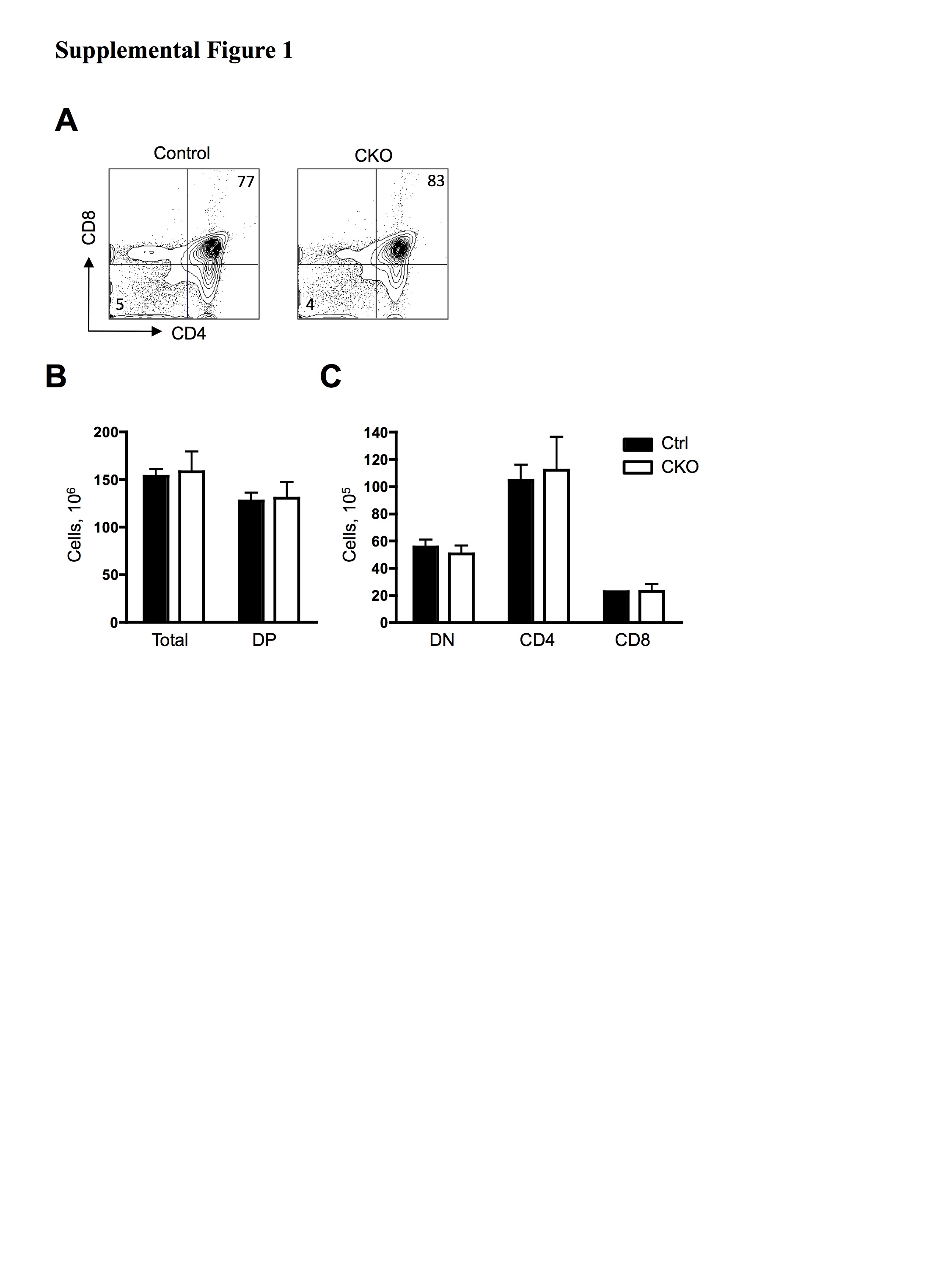

Supplement: Figure S1 — Zfx(fl/y) CD4-Cre mice progress normally through the stages of T cell development. (A) Developing T cell populations in the thymus. Shown are FACS profiles of thymocytes in control and conditional knockout mice; numbers represent the percentage of each population and are representative of over five experiments. (B) Distribution of thymocyte populations. Shown are absolute numbers ± SEM of various thymocyte populations based on expression of CD4 and CD8 as detected by flow cytometry; DP, double positive. Data are representative of two to five mice. (C) Distribution of thymocyte populations. Shown are absolute numbers ± SEM of various thymocyte populations based on expression of CD4 and CD8 as detected by flow cytometry. DN, double negative. Data are representative of two to five mice. [file image_1.jpeg]

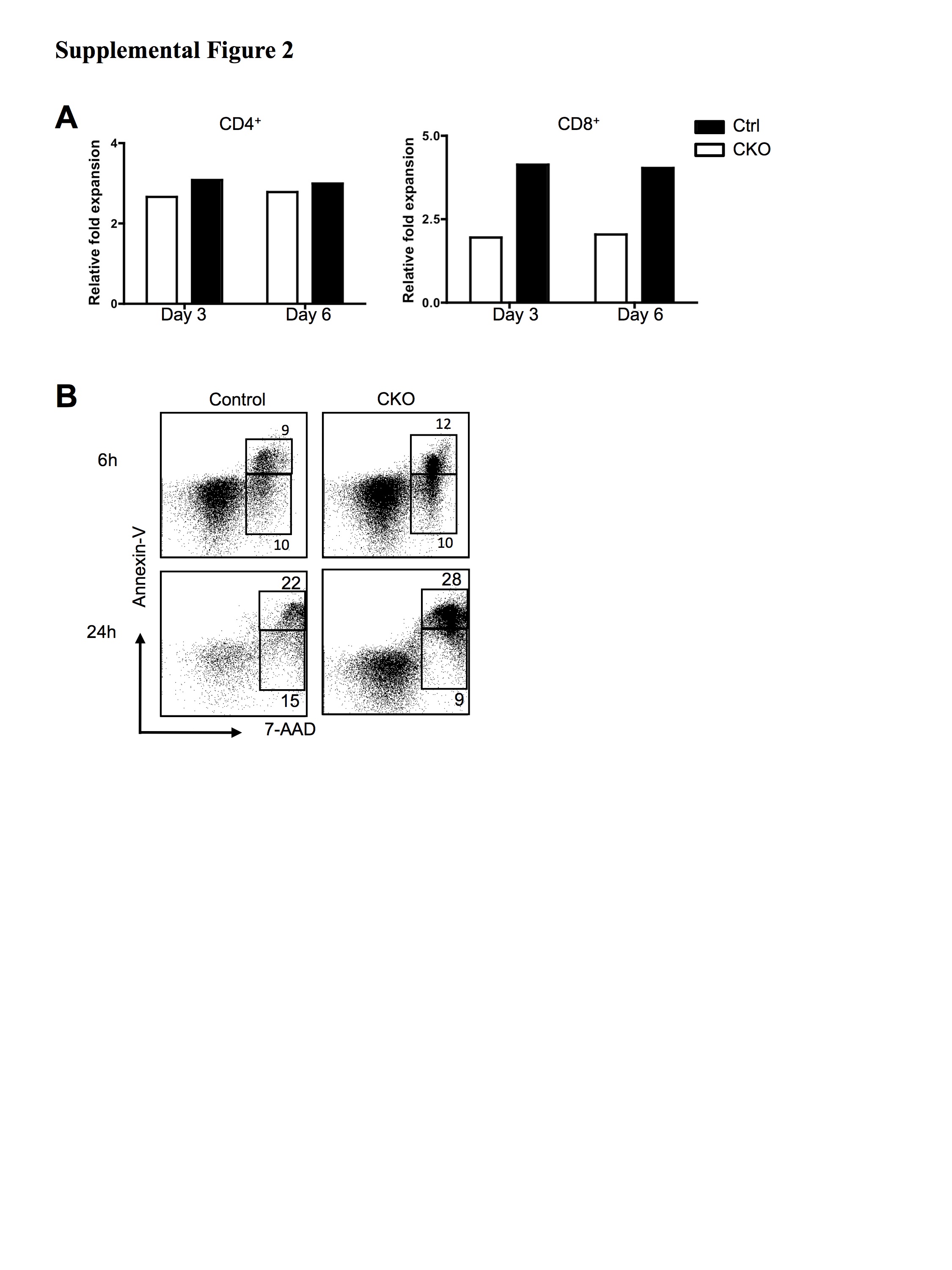

Supplement: Figure S2 — Zfx-deficient T cells have normal response to IL-7 and display normal levels of apoptosis in vitro. (A) In vitro expansion of T cells in response to IL-7. CD62L+ naïve T cells were isolated and maintained in 1 ng/mL IL-7 for 6 days. Shown is the fold expansion of control and conditional knockout (CKO) cells on days 3 and 6, for CD4+ (left) and CD8+ cells (right). Data are representative of more than three independent experiments. (B) In vitro apoptosis. Control and CKO splenic T cells were isolated and maintained in culture for 6 and 24 h, after which, they were stained for Annexin-V and 7-AAD. Numbers represent the percentage of early (Ann-V+7-AAD−) and late (Ann-V+7-AAD+) apoptotic cells; data are representative of two independent experiments. [file image_2.jpeg]

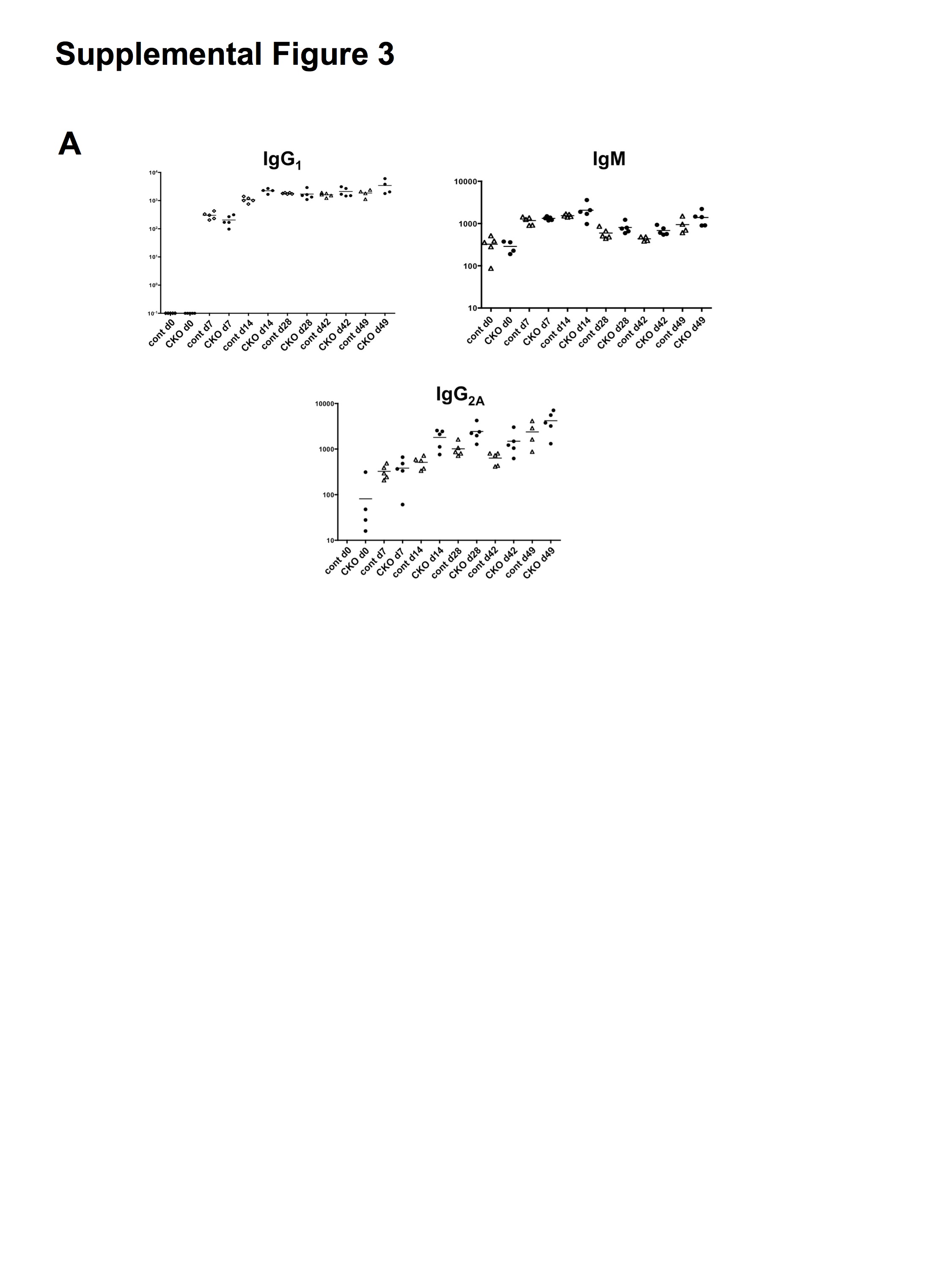

Supplement: Figure S3 — Zfx deficiency has minimal effect on stimulation of B cell antibody production. Zfx-deficient T cells can drive B cell antibody production in vivo. Control (open triangles) and conditional knockout (CKO) (CD4-Cre Zfxflox/y; closed circles) mice were immunized with NP-keyhole limpet hemocyanin (KLH) and antibody titers were measured by ELISA every week post-immunization, for 7 weeks. Mice were boosted with a second dose of NP-KLH on day 42. Shown are relative antibody titers from five control and five CKO mice. [file image_3.jpeg]

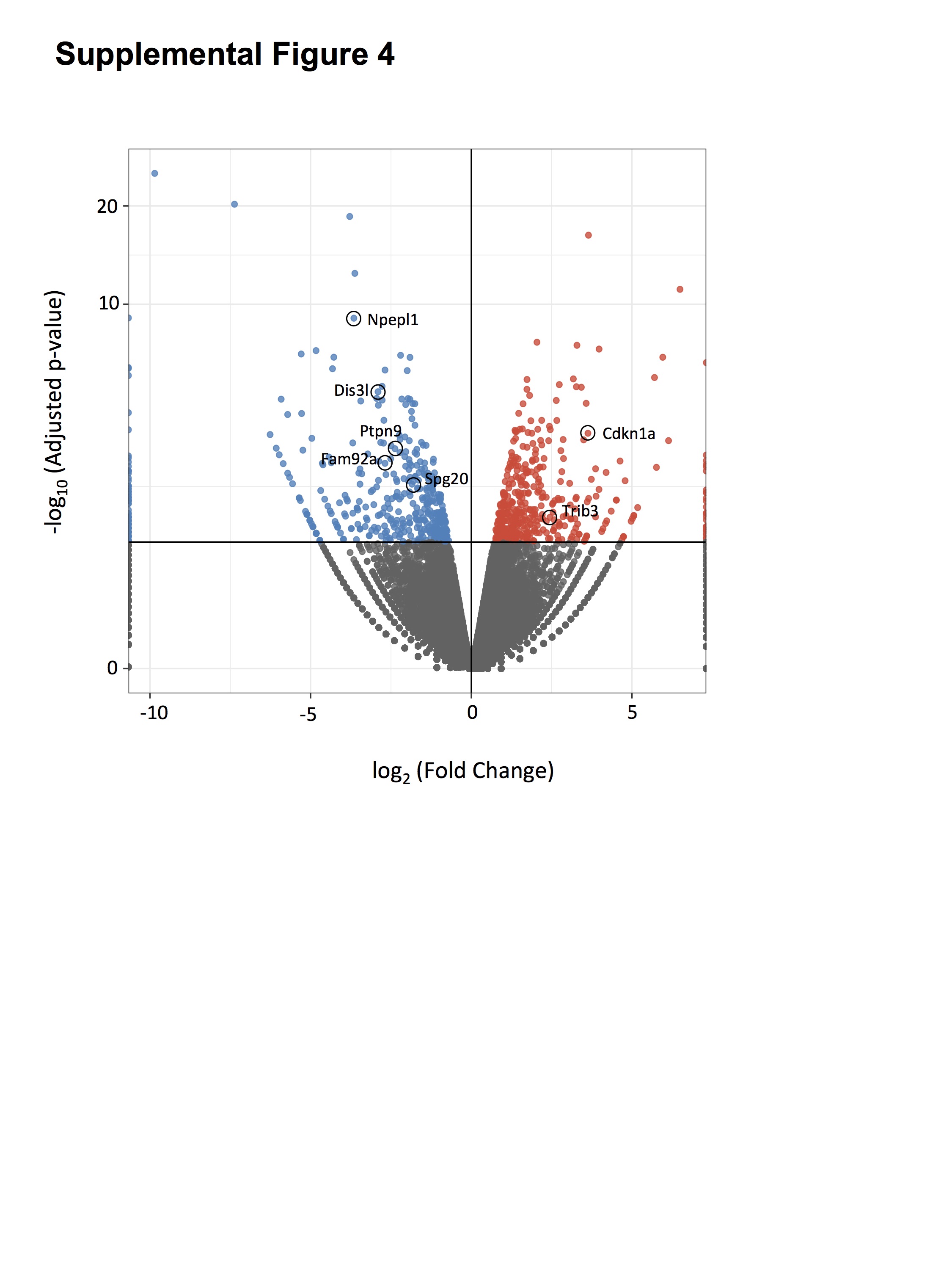

Supplement: Figure S4 — Zfx-deficient T cells simulated in vitro display similar expression defects as unstimulated T cells as well as hematopoietic stem cells. Summary of the RNA-seq results. Volcano plot representation of differential expression analysis of genes in the control versus Zfx conditional knockout T cells. Red and blue points mark the genes with significantly increased or decreased expression, respectively, in control compared to Zfx-null samples. The x-axis shows log2 fold-changes in expression and the y-axis the adjusted P-value. [file image_4.jpeg]
